# Emerging roles for IL-25 and IL-33 in colorectal cancer tumorigenesis

**DOI:** 10.3389/fimmu.2022.981479

**Published:** 2022-10-03

**Authors:** Eric Jou, Noe Rodriguez-Rodriguez, Andrew N. J. McKenzie

**Affiliations:** MRC Laboratory of Molecular Biology, Cambridge, United Kingdom

**Keywords:** IL-25, IL-33, colorectal cancer, mouse model, microenvironment, cytokine

## Abstract

Colorectal cancer (CRC) is the second leading cause of cancer-related death worldwide, and is largely refractory to current immunotherapeutic interventions. The lack of efficacy of existing cancer immunotherapies in CRC reflects the complex nature of the unique intestinal immune environment, which serves to maintain barrier integrity against pathogens and harmful environmental stimuli while sustaining host-microbe symbiosis during homeostasis. With their expression by barrier epithelial cells, the cytokines interleukin-25 (IL-25) and IL-33 play key roles in intestinal immune responses, and have been associated with inappropriate allergic reactions, autoimmune diseases and cancer pathology. Studies in the past decade have begun to uncover the important roles of IL-25 and IL-33 in shaping the CRC tumour immune microenvironment, where they may promote or inhibit tumorigenesis depending on the specific CRC subtype. Notably, both IL-25 and IL-33 have been shown to act on group 2 innate lymphoid cells (ILC2s), but can also stimulate an array of other innate and adaptive immune cell types. Though sometimes their functions can overlap they can also produce distinct phenotypes dependent on the differential distribution of their receptor expression. Furthermore, both IL-25 and IL-33 modulate pathways previously known to contribute to CRC tumorigenesis, including angiogenesis, tumour stemness, invasion and metastasis. Here, we review our current understanding of IL-25 and IL-33 in CRC tumorigenesis, with specific focus on dissecting their individual function in the context of distinct subtypes of CRC, and the potential prospects for targeting these pathways in CRC immunotherapy.

## Introduction

CRC affects the colon or rectum, and is third on the list of most common cancers globally (1). Despite improvements in early detection and diagnosis, CRC remains the second leading cause of cancer-related mortality worldwide, underlying ~10% of total cancer deaths. It is estimated that this incidence may increase by a further 60% by 2030 (2). CRC is a heterogenous disease encompassing different subtypes. The majority (~80%) of sporadic human CRCs have causative loss-of-function mutations in the *adenomatous polyposis coli* (*APC*) tumour suppressor gene, and progress over several years from benign polyps to metastatic disease (3). Other rarer subtypes of CRC, such as microsatellite-instability-high (MSI-high) CRC (~15% of CRC) primarily driven by mismatch-repair-deficiency (4), and colitis-associated colorectal cancer (CAC) secondary to inflammatory bowel disease (IBD) (1-2% of CRC) (5, 6), have distinct driver mutations and disease etiologies.

The intestinal tract is inhabited and visited by huge numbers of immune cells, and harbors the largest proportion of the host immune system (7). With its continuous exposure to the external environment during the processing of dietary intake, and its hosting of an enormous and diverse microbiome, the intestinal immune compartment must protect the host from harmful pathogens, while maintaining tolerance to self-tissues, dietary antigens, and hosting commensal microorganisms. Through embryonic development (Stras et al., 2019) and subsequent exposure to external environmental stimuli after birth (8) the intestinal immune system adapts continuously during its education by host and environmental factors (9–12), and develops a highly specialised compartmentalisation required for its proper function (13, 14). A complex array of immune interactions between secreted molecules and cell surface receptors across a diverse collection of epithelial, stromal and immune cells help maintain immune homeostasis and tolerance during the steady state, but can also react rapidly to infection to orchestrate protective immunity (15, 16). Dysregulation of this intricate system has been implicated in a wide range of pathologies including autoimmunity and cancer (17–19).

IL-25 and IL-33 are epithelial cell-derived cytokines that play important roles in intestinal immunity. Both IL-25 and IL-33 are potent activators of type-2 immunity, which is critical in parasite helminth expulsion, and promoting tissue repair and regeneration (20–22). Nevertheless, recent studies suggest that there are important differences in their tissue-specific functions (23). For example, in the intestines, IL-33 and IL-25 are produced by non-overlapping cellular sources, and have distinct regulatory mechanisms behind their storage and release (24). Furthermore, IL-33 and IL-25 mediate effector functions through distinct receptors, with differential expression of these receptors on shared and distinct cell types. In the past decade, emerging studies have begun to uncover the previously unappreciated role of IL-25 and IL-33 in CRC disease pathogenesis and in shaping the tumour niche, with distinct effects specific to the different subtypes of CRC.

In this review, we outline how the dynamics of intestinal immune responses driven by IL-25 and IL-33 impact CRC development in the context of CRC subtypes, with reference to how different mouse models provide insight (sometimes imperfect) into the immune reactions in the tissues in different subtypes of CRC. Consequently, we examine how targeting the inhibition of IL-25, IL-33 and type 2 intestinal immunity, to release the brakes on type 1 anti-cancer immunity, may be beneficial in certain CRC subtypes, but detrimental in others.

## Overview of IL-25 and IL-33 in CRC subtypes

### Cytokine biology

**Figure 1:**
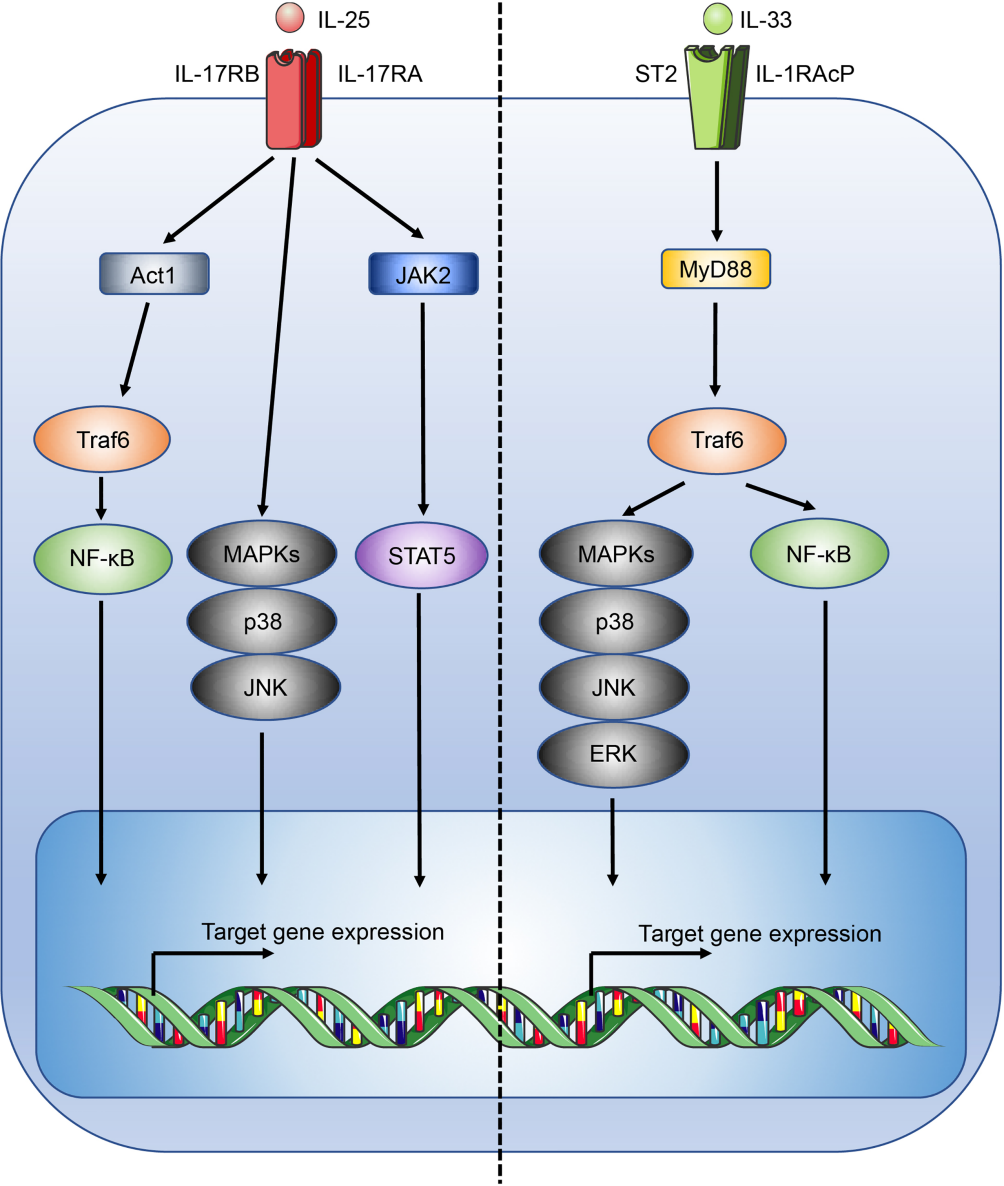
Overview of the IL-25 and IL-33 signalling pathways. Figure depicts the IL-25 (left) and IL-33 (right) signalling pathways. Act1, CIKS; c-Jun N-terminal kinase, JNK.

IL-25, also known as IL-17E, is part of the IL-17 family of cytokines. IL-25 binds a heterodimeric receptor, composed of interleukin-17 receptor A (IL-17RA) and interleukin-17 receptor B (IL-17BR) (25), which is expressed by Th2 cells, Tregs, ILC2s, NKT cells, and signals through Act1, TRAF6, NF-κB, MAPK and STAT5-mediated pathways (**Figure 1**) (26–29). In the intestines, IL-25 is secreted by specialised epithelial chemosensory cells named tuft cells that express the marker doublecortin-like kinase 1 (DCLK1) (30), which are estimated to constitute ~1 - 7% of the intestinal epithelial compartment in mice, with variations possibly due to the effects of the microbiota on the mucosal immune system (31). Functionally and phenotypically similar cells exist in other tissues but they have received different names, such as brush cells in the airways (32).

Conversely, IL-33 is constitutively expressed at high levels by a wide range of cell types including epithelial cells at mucosal barriers, endothelial cells, fibroblasts, and stromal cells (33, 34). While IL-33 expression within the intestinal epithelial compartment is widespread, it is not, unlike IL-25, produced by tuft cells (30, 35). The distinct expression pattern of IL-25 and IL-33 is reflective of the structural and functional differences of the two cytokines. IL-33 is a member of the IL-1 family of cytokines and lacks a secretory signal peptide (36) and instead contains a nuclear localisation sequence at its N-terminus, allowing storage in the nucleus where it is thought to act as a transcriptional repressor (37, 38). Without a secretory signal sequence IL-33 is not secreted like conventional cytokines such as IL-25, and is instead released upon cell damage (39), in line with its postulated role as an “alarmin”. To avoid inappropriate inflammation, apoptotic turnover of epithelial cells activates caspases that inactivate IL-33 (39). Notably, a recent report has identified a role for the pore-forming protein gasdermin C in the atypical secretion of IL-33 by intestinal epithelial cells (40). During inflammation, IL-33 expression is further upregulated, and can be processed extracellularly by mast cell chymases and tryptases into mature forms, increasing IL-33 activity by 10 - 30 fold (41). Subsequently, inactivation of IL-33 in the extracellular space occurs through oxidation (42).

IL-33 mediates its immune functions through a heterodimeric receptor complex comprising the IL-33 specific receptor ST2 (interleukin 1 receptor-like 1, Il1rl1), coupled with the IL-1 receptor accessory protein (IL-1RAcP) coreceptor (43, 44). ST2 is typically expressed by a wide range of immune cells involved with type 2 immunity, including ILC2s, mast cells, basophils, dendritic cells, eosinophils, M2-like macrophages, and Th2 cells (45). Upon IL-33 binding to ST2, signalling occurs through the recruitment of the adaptors MyD88 and TNF receptor associated factor 6 (Traf6) leading to the activation of mitogen-activated protein kinases (MAPKs) and NF-κB (**Figure 1**) (46). In the lungs IL-33 potently stimulates airway contraction, mucus production, and goblet cell hyperplasia through early induction of IL-13 in ILC2s, and plays important roles in allergic diseases and asthma (47, 48). While immune cells associated with type 2 immunity may co-express IL-17BR and ST2 allowing a degree of potentiation in the induction of a type 2 immune response, IL-33 and IL-25 are shown to induce distinct activation phenotypes, for example in ILC2s, indicating functional divergence (47, 49).

While best characterised for their role in type 2 immunity, IL-25 and IL-33 have additional functions in modulating other arms of immunity. IL-33 has been shown to also support Th1, Th17 and Treg responses (50–52). Activation of innate NK cells, and adaptive Th1 and CD8^+^ T cells by the classical type 1 immunity polarising cytokine IL-12, induces the upregulation of the IL-33 receptor ST2, and IL-33 exposure greatly increases their ability to produce IFNγ, the signature effector cytokine of classical type 1 responses. It is postulated that IL-33 supports Th17 immunity through mast cells, and activated Th17 cells have recently been shown to express ST2 in the gut (53). Conversely, IL-25 has been shown to suppress both Th1 and Th17 immunity in inflammatory conditions (54, 55). IL-33 also plays essential roles in the differentiation and maintenance of ST2^+^ Tregs in the intestines, supporting their function during inflammation (52). In line with the wide range of functions, both IL-25 and IL-33 have been implicated in a broad range of diseases. IL-25 signaling is proposed to be involved in infection, asthma and allergy, psoriasis, autoimmunity including inflammatory bowel disease (IBD), rheumatoid arthritis, multiple sclerosis, Sjögren’s Syndrome and cancer (56–59). Similarly, IL-33 has been implicated in the pathogenesis of chronic obstructive pulmonary disease (COPD), asthma, IBD, obesity, diabetes, cardiovascular and musculoskeletal diseases, and cancer (60, 61).

### CRC subtypes

**Figure 2:**
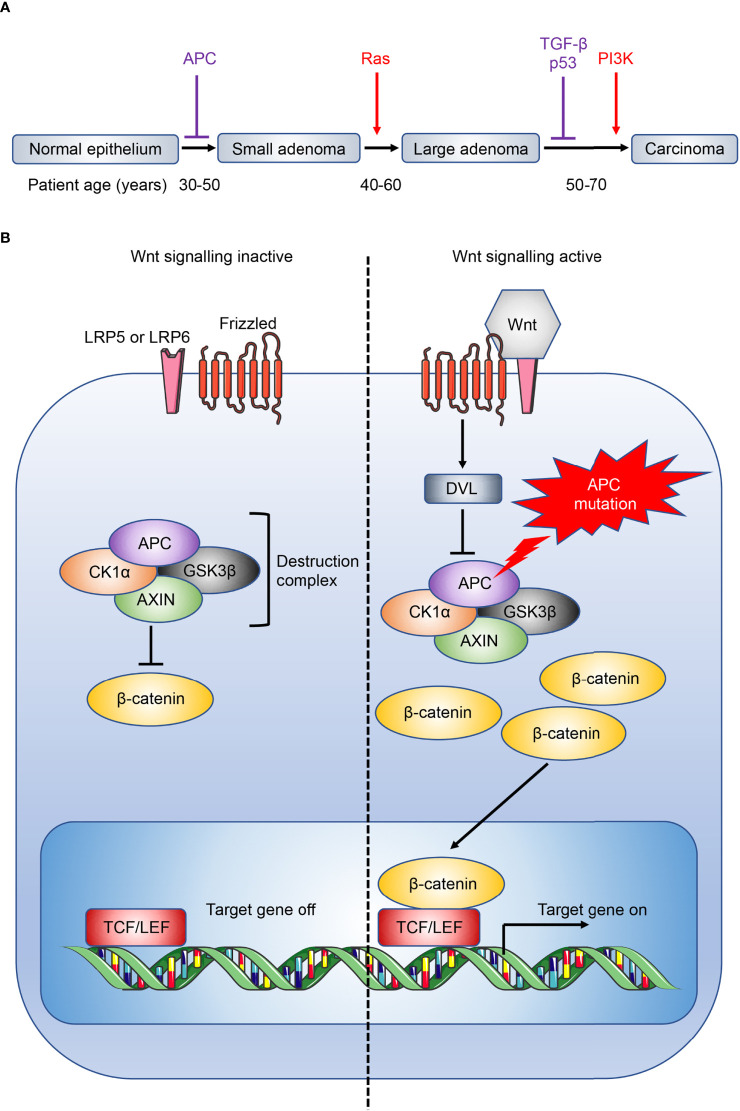
Overview of CRC development and Wnt signaling pathway. **(A)** CRC development is associated with a gradual accumulation of genetic alterations involving the loss of tumour suppressors (purple) or gain of oncogenic function (red). Loss of the tumour suppressor adenomatous polyposis coli (APC) facilitates the initial transition from normal epithelium to adenomas. Patient age when the driver genes are commonly mutated are also shown (66). Ras, rat sarcoma virus; TGF-β, transforming growth factor-β; p53, tumour protein p53; PI3K, phosphatidylinositol 3-kinase. **(B)** The destruction complex, consisting of APC, AXIN, casein kinase 1α (CK1α) and glycogen synthase kinase 3β (GSK3β), induces the continuous degradation of β-catenin in the absence of Wnt signaling. The destruction complex is disassembled when Wnt ligand binds to its receptor frizzled and coreceptor lipoprotein receptor-related protein 5 or 6 (LRP5 or LRP6 respectively), or when APC is mutated, leading to the accumulation and nuclear translocation of β-catenin, and downstream Wnt signaling (67). DVL, dishevelled; TCF/LEF, T cell factor/lymphoid enhancer factor.

Despite recent advances, immunotherapy in CRC patients is largely disappointing (62), in particular for *APC* gene-mutation initiated, microsatellite-stable (MSS) CRC (63, 64) which do not respond to checkpoint inhibitors (4, 65). Tumour vaccines and adoptive T cell transfers have also shown little efficacy in these patients (17). APC inhibits the Wnt signaling pathway (**Figure 2**), and loss-of-function mutations in APC lead to accumulation and nuclear translocation of β-catenin, resulting in aberrant upregulation of Wnt signaling and colorectal tumour initiation (3, 63, 68, 69). Currently, immunotherapy in CRC is largely limited to MSI-high CRC where checkpoint inhibitors show efficacy (4, 70). The differential efficacy of checkpoint inhibitors against MSI-high and MSS CRC reflects the distinct immune microenvironment in different subtypes of CRC (71). MSI-high tumours have defects in the DNA mismatch-repair pathway, most commonly due to epigenetic silencing of the *MutL homolog 1* (*MLH1*) gene or inactivation of *MSH2*, and consequently accrue extensive genomic mutations that lead to the expression of neoantigens by the tumours, and their immune recognition by neo-antigen specific cytotoxic T cells (72, 73). This immunogenicity underlies the striking efficacy of immunotherapeutic checkpoint inhibitors in these patients (KEYNOTE-177) (74). In addition, CAC secondary to IBD is a rarer subtype of CRC that is mutationally distinct from other human CRC subtypes, with whole-exome sequence analysis revealing only 13% incidence of *APC* mutation in CAC (6, 63). Importantly, immuno-profiling of CAC and sporadic CRC samples showed different immune patterns consistent with distinct disease phenotypes, with decreased immune cells that resemble CD8^+^ T cells and regulatory T cells (Tregs) in CAC compared to sporadic CRC (75). Studies investigating the role of IL-25 and IL-33 have found conflicting results across the various mouse models that resemble distinct human CRC subtypes, where they may either promote or inhibit tumorigenesis depending on the CRC subtype through different downstream immune mechanisms. As such, it is important to recognize that findings in a specific CRC subtype may not extend to encompass all CRC. A range of mouse models have been developed to model different subtypes of CRC, and these have been discussed in depth by recent reviews (76, 77).

### IL-25 in CRC

**Figure 3:**
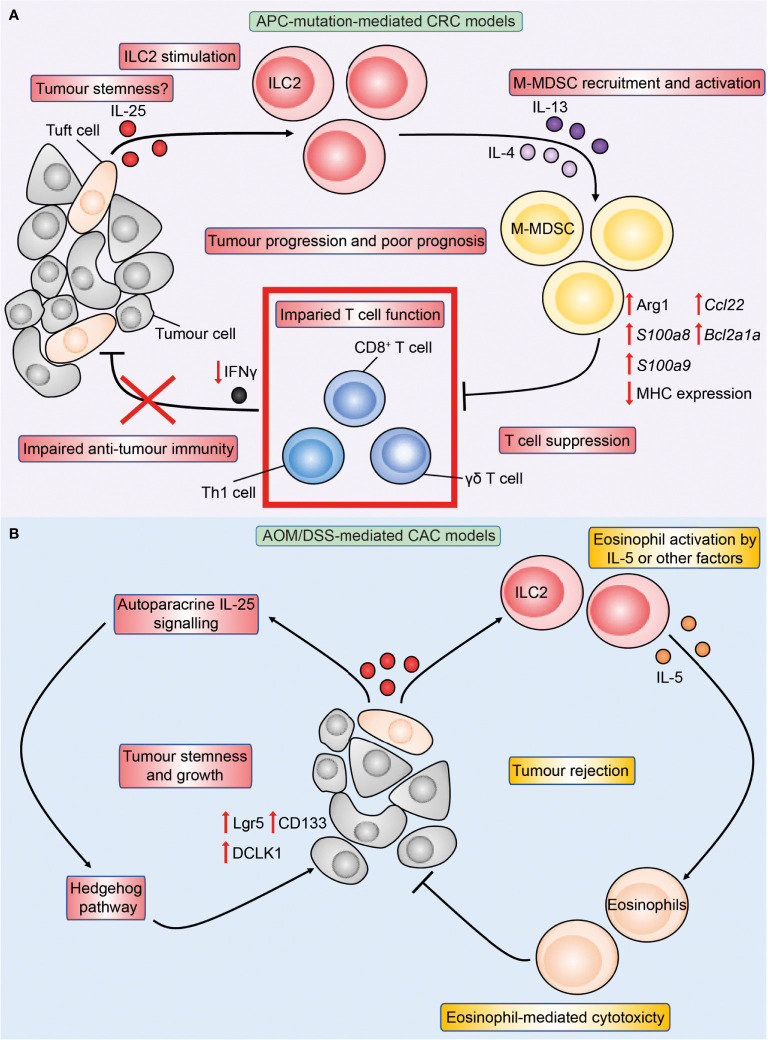
Overview of IL-25 in APC-mutation-mediated CRC and colitis-associated cancer (CAC) models. Figure depicts the immune-regulatory role of IL-25 characterised in preclinical models of APC-mutation-mediated CRC **(A)** and CAC **(B)**. **(A)** In the intestinal tumour microenvironment, DCLK1^+^ tuft-like cells are the main source of IL-25, and the latter may in turn promote tumour stemness. IL-25 activates tumour IL-25R^+^ ILC2s to produce IL-4 and IL-13, which increases arginase 1 (Arg1) expression in tumour M-MDSCs leading to enhanced M-MDSC suppressive capacity. M-MDSC-mediated T cell suppression reduces T cell proliferation and IFNγ production, leading to impaired anti-tumour immunity and tumour progression. **(B)** In the AOM/DSS model of CAC, IL-25 reduces tumour burden through eosinophils. This may be *via* ILC2-derived IL-5 or other yet-to-be identified factors promoting eosinophil activation and cytotoxicity against tumours in a CD8^+^ T cell-independent manner. Conversely, IL-25 may act in an autoparacrine manner to promote tumour stemness characterised by upregulation of stem markers Lgr5, CD133 and DCLK1 downstream IL-25 signaling in a Hedgehog pathway-dependent manner.

The prominent roles of IL-33 and IL-25 in modulating the intestinal immune response made them attractive candidates for investigation in CRC and as potential targets for immunotherapy. In the past decade many studies have shown that IL-33 modulates CRC pathogenesis, while in more recent years the roles of IL-25 in CRC are increasingly being recognised. IL-25 has been found to be highly expressed in human CRC by two histopathological studies (78, 79). Analysis of IL-25 expression in healthy colons and CRC showed comparable levels in one report (78), while a more recent study found IL-25 expression to be elevated in human CRC tumours compared to the adjacent normal gut (80). Similar to the homeostatic intestine and mirroring IL-25 expression during parasitic infection (30), DCLK1^+^ tuft cells were found to be the main source of IL-25 in tumours in mouse models of APC-mutation-mediated CRC (*Apc* ^1322T/+^) (81) and azoxymethane (AOM)/dextran sodium sulphate (DSS)-mediated model of colitis associated cancer (CAC) (80), indicating that IL-25 expression by tuft cells is conserved across different subtypes of CRC. Recently, analysis of the publicly available gene expression databases found that high *IL25* expression by CRC tumours was associated with reduced CRC patient disease-free survival (81). Others have shown that human patients with high CRC IL-25 expression have worse 5-year overall survival than stage IV CRC patients with low IL-25 expression (80), indicating that IL-25 may contribute to CRC disease pathogenesis. As unselected CRC patients were assessed in these studies, the majority of patients would have sporadic APC-mutation-mediated CRC (85% of total CRC). In *Apc* ^1322T/+^ mice, genetic deficiency of IL-25 reduced intestinal tumour burden and virtually doubled survival, and antibody-mediated blockade of IL-25-signalling similarly reduced CRC (81). These studies indicate a pro-tumoral role of IL-25 in autochthonous APC-mutation-mediated CRC. Conversely, IL-25 has conflicting roles in CAC. Genetic deficiency of IL-25 reduced CAC tumour burden and prolonged survival in one study (80), however others reported that antibody-mediated blockade of IL-25 instead enhanced CAC (82). As both studies employed the AOM/DSS-model of CAC, this suggests that congenital deficiency of IL-25 may have distinct effects compared to acute blockade in CAC. Finally, IL-25 injections inhibited heterotypic subcutaneous tumour growth across a range of human cancer cell lines in immunocompromised T cell-deficient nude mice, including a HT-29 CRC cell line (83). Therefore, the role of IL-25 in CRC is likely dependent on the disease stage and CRC subtype (**Figure 3**), and hence may be reflected differently in the mouse model investigated.

### IL-33 and CRC

In CRC, IL-33 is predominantly expressed by tumour epithelial cells and is upregulated in both human and mouse (*Apc* ^min/+^) intestinal tumours compared to the adjacent normal gut (84). IL-33 expression has similarly been detected in the AOM/DSS model of CAC (85). Unlike tumour *IL25* gene expression which is associated with poor survival in CRC patients, *IL33* expression was not found to correlate with differential CRC patient survival (81, 86). While an earlier study found no association of tumour *ST2* expression with CRC patient survival (86), two recent studies showed that CRC patients who have increased tumour *IL1RL1* expression and high densities of ST2-positive cells showed reduced overall survival (87, 88), indicating that ST2^+^ cells may contribute to CRC pathogenesis. Conversely, others have found that decreased *IL33* and *ST2* expression is associated with advanced human CRC and poor survival respectively, and ST2^+^ CRC showed reduced vascular invasion and lymph node metastasis (89, 90), indicating that the role of IL-33 and ST2 in CRC is likely complex and may depend on the CRC subtype and disease stage.

**Figure 4:**
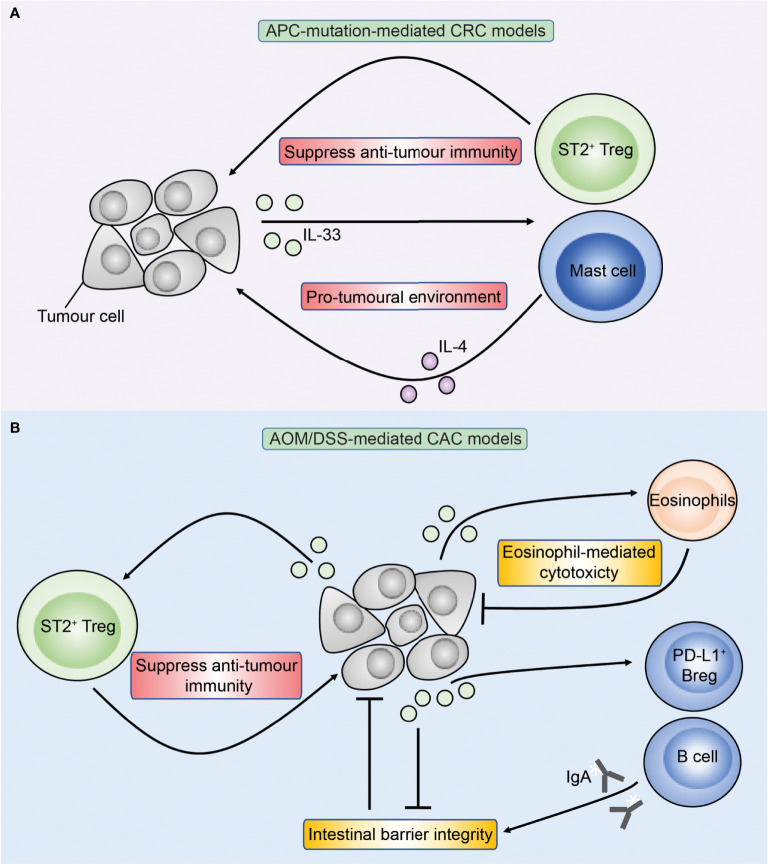
Overview of IL-33 in APC-mutation-mediated CRC and colitis-associated cancer (CAC) models. Figure depicts the role of IL-33 in preclinical models of APC-mutation-mediated CRC **(A)** and CAC **(B)**. **(A)** In APC-mutation-mediated CRC models, IL-33 is proposed to promote tumorigenesis through activating ST2^+^ Tregs and mast cells which may have pro-tumoral properties through suppressing anti-tumour T cells and production of IL-4 respectively. **(B)** In AOM/DSS-mediated models of CAC, IL-33 can similarly promote tumorigenesis through ST2^+^ Tregs, but also *via* direct action on ST2^+^ epithelial tumour cells compromising the intestinal barrier resulting in colitis and CAC. Conversely, IL-33 may inhibit CAC through sustaining intestinal barrier integrity *via* stimulating B cells to produce IgA, and also *via* direct activation of anti-tumoral eosinophils.

Studies of IL-33 in mice have similarly demonstrated conflicting results where IL-33 may either promote or inhibit CRC (**Figure 4**) (84, 90–92). In *Apc*-mutant mice, reports are consistent with IL-33 exerting a pro-tumoral role (81, 84, 91). Genetic deficiency of IL-33 or antibody-mediated blockade of ST2 led to fewer tumours (81, 84) while transgenic overexpression of IL-33 specifically in intestinal epithelial cells *via* the villin promoter enhanced tumorigenesis in *Apc* ^min/+^ mice (91). In a separate model, through IL-33-mediated upregulation of COX-2 in tumour cells, IL-33 transgenic mice with subcutaneous implants of MC38 tumours showed increased growth and proliferation markers compared to wild type mice, which was abrogated upon treatment with a selective COX2 inhibitor (93). Meanwhile, the role of IL-33 in CAC is more contentious. Genetic deficiency of ST2 (*St2* ^-/-^) reduced tumour burden in the AOM/DSS mouse model of CAC, and was associated with improved intestinal barrier integrity reducing bacterial translocation and inflammation (90). Accordingly, inhibition of the epidermal growth factor receptor (EGFR) *via* gefitinib in AOM/DSS mice led to decreased tumour burden, and this correlated with a reduction in intestinal epithelial cell *Il33* expression which the authors attributed to for the pro-tumoral effect of EGFR signalling (85). However, further investigation is required to determine how much of the reduction in tumour burden can be attributed to a reduction in *Il33* expression, rather than to a direct effect of EGFR inhibition. Conversely, in another study genetic deficiency of IL-33 significantly enhanced tumour burden in AOM/DSS-treated mice (92). Therefore regulation of IL-33 *in vivo* is multi-layered and highly complex, and its impact on CRC tumorigenesis may be affected by the overall tumour microenvironment and the presence of different immune cell populations, which may underlie the conflicting results.

Intriguingly, while both IL-25 and IL-33 expression is elevated in human CRC compared to the adjacent normal gut, in some studies expression of both cytokines was found to be highest in early colorectal adenomas compared to adenocarcinoma (79, 86). Therefore, both IL-25 and IL-33 and downstream effector functions may be affected by temporal factors during CRC disease progression providing additional complexity. Various mechanisms have been proposed on how IL-25 and IL-33 may either promote and inhibit CRC pathogenesis, involving many different pathways associated with CRC development. These will be discussed in turn below.

## Immune modulation by IL-25 and IL-33 in CRC

As potent orchestrators of intestinal immunity, IL-25 and IL-33 can modulate CRC pathogenesis through various downstream immune cells. Despite their overlapping function in asthma and inflammatory diseases associated with type 2 immunity, existing studies suggest that IL-25 and IL-33 elicit largely distinct immune pathways in CRC. Specifically, IL-25 has been shown to preferentially affect ILC2s and myeloid-derived suppressor cells (MDSCs) in CRC (81), while IL-33 exerts a more dominant effect on Th2 cells, Tregs, and mast cells (84, 87, 88, 91, 92, 94–96), although both can modulate eosinophils (82, 97). Adding to the complexity, the downstream immune response elicited by IL-25 and IL-33 is also dependent on the CRC subtypes, in line with the distinct immune profiles in the latter.

### IL-25 and ILC2s

Until recently, the role of IL-25 in cancer has been largely speculative and few studies to date investigated the downstream immune mechanisms of IL-25 in CRC. Our group recently reported that IL-25 can promote CRC through ILC2s. ILC2s are innate immune cells that orchestrate type-2 immunity by providing an early innate source of the type-2 cytokines IL-4, IL-5, IL-9, and IL-13 (16, 21, 98, 99) to induce intestinal parasitic helminth worm expulsion (21, 99) and sustain tissue repair and regeneration (20–22). ILC2s can express the receptors for IL-25 and IL-33, but expression may vary dependent on the tissue immune microenvironment and inflammatory mediators. Intestinal ILC2s express higher levels of IL-17BR compared to those found in other tissues such as the bone marrow, skin, fat and lung, while expression of the IL-33 receptor ST2 is minimal (100). This suggests that IL-25 may play a more dominant role in stimulating ILC2s in the intestinal environment.

**Figure 5:**
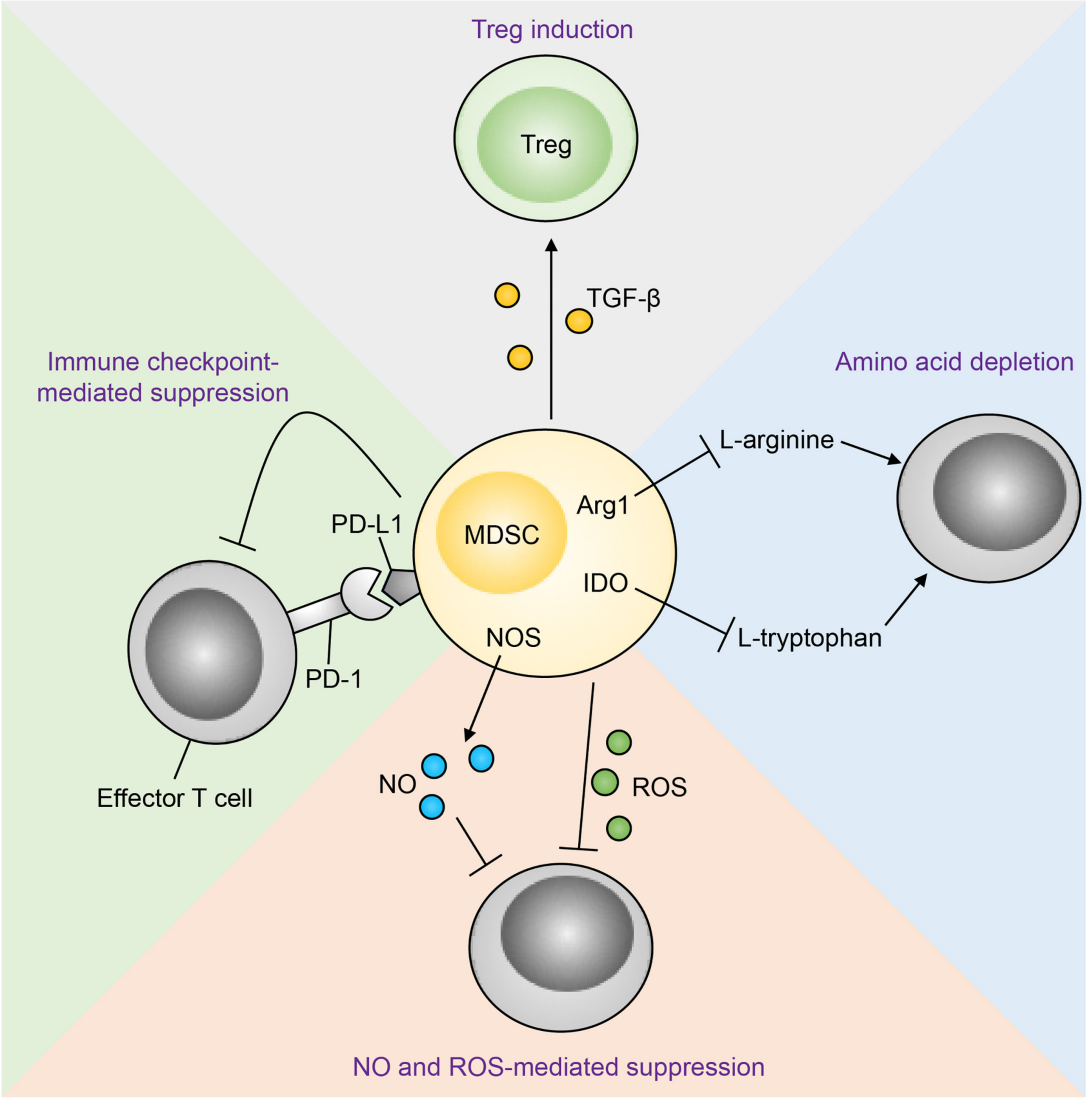
Mechanisms of MDSC-mediated T cell suppression. Immunosuppression of effector T cells by MDSCs occurs through multiple mechanisms. MDSC-derived nitric oxide (NO) and reactive oxygen species (ROS) inhibit T cell effector function and promote apoptosis. MDSCs can also suppress effector T cells through amino acid depletion MDSCs express arginase 1 (Arg1) and indoleamine-pyrrole 2,3-dioxygenase (IDO), which deplete the amino acids L-arginine and L-tryptophan respectively in the tumour microenvironment. Metabolic suppression of T cells ensues as these amino acids are essential for T cell effector function. In addition, MDSCs express programmed death-ligand 1 (PD-L1), which interacts with its receptor programmed cell death protein 1 (PD-1) on T cells, resulting in T cell functional exhaustion. Finally, MDSC-derived transforming growth factor-β (TGF-β) converts T cells to a regulatory phenotype (Tregs). NOS, nitric oxide synthase.

Importantly, recent studies have reported an abundance of ILC2s in human CRC (101–103), and that tumour-infiltrating ILC2s in human CRC express IL-17BR, which is mirrored in APC-mutant mice (81). Ablation of IL-25 signaling reduced tumour load, ILC2s and extended survival of IL-25-deficient *Apc* ^1322T/+^ mice. ILC2-deficient mice (*Rora* ^f/f^ *Il7r* ^Cre/+^ *Apc* ^1322T/+^ mice) also developed fewer tumours, which correlated with increased frequencies of Th1 cells and CD8^+^ T cells, and a reduction in MDSCs. Th1 and CD8^+^ T cells are potent producers of IFNγ, which have been shown to be anti-tumoral by several groups in APC-mutant mouse models (104, 105). Critically, rIL-25 treatment increased tumour burden in ILC2-replete but not ILC2-deplete *Apc* ^1322T/+^ mice, indicating an essential role for ILC2 in driving IL-25-mediated CRC tumorigenesis (81). Importantly, rIL-33 treatment was able to increase tumour burden independently of ILC2s in the same model. Furthermore, IL-25-signalling blockade *via* anti-IL17BR antibody administration in *Apc* ^1322T/+^ mice reduced ILC2s and pro-tumoral MDSCs while increasing IFNγ^+^ CD4^+^ and CD8^+^ T cells in colonic adenocarcinomas, indicating a potential therapeutic intervention to block the pro-tumoral role of the IL-25-ILC2-monocytic MDSC (M-MDSC) axis in CRC. MDSCs mediate suppression of anti-tumour adaptive T cells through a plethora of mechanisms including expression of checkpoint ligands such as programmed death-ligand 1 (PD-L1) (**Figure 5**) (106–114). Mechanistically, ILC2-derived IL-4 and IL-13 in response to IL-25 signaling activated tumour M-MDSCs derived from *Apc* ^1322T/+^ mice to express Arginase 1 (Arg1) and suppress CD8^+^ T cells (81) . In human CRC samples, ILC2s positively correlated with M-MDSCs, while negatively correlating with Th1 cells and CD8^+^ T cells. Many independent studies have confirmed Th1 and CD8^+^ T cell infiltration to be amongst the strongest positive prognostic factors for improved CRC patient survival across all stages of disease (115–121). These results indicate that an IL-25-ILC2-M-MDSC pathway acts independent of IL-33-mediated pathways to produce a cancer-permissive immune niche in APC-mutation-mediated CRC (**Figure 3**), and may potentially be targeted in patients. By contrast, ILC2-deficiency increased tumour burden in an AOM/DSS model of CAC indicating an anti-tumoral role of ILC2s. Thus, differential mechanisms or interactions between the IL-25-ILC2 axis and pro-tumoral MDSCs may exist in CAC (122).

### IL-33 and MDSCs

IL-33 has been shown to sustain MDSCs in human and mouse breast cancer (123), and there is some evidence that IL-33 may also affect MDSC recruitment in CRC. In an orthotopic caecal implant model of CRC using MC38 cells, overexpression of IL-33 in tumour cells led to increased liver metastasis that was associated with an increased recruitment of CD11b^+^Gr1^+^ MDSCs and tumour angiogenesis (124). However, a more recent study found no effect of IL-33 treatment on tumour MDSC frequency in a subcutaneous CT26 implant model of CRC (97). These discrepancies may be due to the different site of implant or CRC cell line used. MC38 cells have a higher accumulation of mutations than CT26 cells (125) and mutational load is known to affect tumour immunogenicity and downstream immune mechanisms (126). Equally the differing immune response tendencies between the two mouse strains BALB/c (CT26) and C57BL/6 (MC38) may also contribute to the differences observed. Critically, cell line implant models do not retain the original tumour architecture and cellular complexity, and CT26 and MC38 models do not harbor mutations in *Apc* (125, 127). Whether IL-33 modulates MDSCs in autochthonous APC-mutation-mediated CRC or CAC, allowing potential functional interaction with the IL-25-ILC2 axis in CRC through MDSCs, remains to be established.

### IL-33 and mast cells

Studies in APC-mutant mice found that decreased tumour burden upon IL-33-deficiency (but not IL-25-deficiency) is associated with reduced tumour mast cells (81, 84), suggesting a potential pro-tumoral role downstream IL-33 signalling. Indeed, mast cells were found to be increased in intestinal tumours from *Apc* ^Δ468^ mice, and depletion of mast cells through the generation of chimeric mice reduced tumour development (128). However, another study found that genetic deficiency of the atypical chemokine receptor 2 (*Ackr2* ^-/-^) in *Apc* ^min/+^ mice led to reduced tumour burden, which the authors attributed to an increase in mast cells that can recruit CD8^+^ T cells through leukotriene B_4_ (LTB_4_) (129). Mast cells may exert anti-tumoral properties in CAC. In an AOM/DSS model of CAC, deficiency in hematopoietic prostaglandin D synthase (H-PGDS) exacerbated tumorigenesis, and adoptive transfer of mast cells deficient in H-PGDS, but not wild type mast cells, into mast cell-deficient (*Kit* ^w-sh/w-sh^) mice exacerbated tumorigenesis (130). It is well-established that IL-33 promotes mast cell activation (131), and as discussed above both IL-33 and mast cells can either promote and inhibit CRC. In human intestinal tumours, mast cells are increased in adenomas and CRC compared to the adjacent normal gut (128, 132, 133), and high tumour mast cell infiltration has been associated with reduced (133) or improved (134) survival in CRC patients. It is recognised that mast cell function in cancer can be tumour stage dependent, for example in promoting early but not late stage prostate cancer growth through MMP9 production (135). Similarly, human CRC *IL33* and *ST2* expression varies according to tumour stage (89, 90). Whether differential IL-33 expression during the stages of tumour progression affect downstream mast cell function, and whether mast cells directly contribute to the tumour-modulatory effects of IL-33 in CRC remains to be established. Mechanistic studies that directly assess the role of mast cells downstream of IL-33 in the context of CRC are required.

### IL-33 and Th2 cells

Genetic overexpression of IL-33 led to increased tumour burden and expression of *Il4*, *Il5* and *Il13* in Th2 cells, and this correlated with increased tumorigenesis in the *Apc* ^min/+^ mice (91), while genetic deficiency of IL-33 reduced overall tumour *Il4* and *Il13* expression (84). On the other hand, IL-25-deficiency had no effect on Th2 cell frequency in *Apc* ^1322T/+^ mice, and instead acts *via* modulating ILC2 numbers and their production of IL-4 and IL-13 (81). Collectively, these studies suggest that IL-25-mediated activation of ILC2s, and IL-33-mediated stimulation of Th2 cells, may act as independent parallel pathways to induce the production of IL-4 and IL-13 leading to the activation of MDSCs to suppress anti-tumour T cells and promote CRC.

### IL-33 and Tregs

Studies suggest that IL-33 may modulate CRC tumorigenesis through Tregs. Like MDSCs, Tregs are immunosuppressive cells that can counteract an anti-tumoral immune response, and hence are traditionally associated with pro-tumoral functions (136). In a CT26 subcutaneous model of CRC, exogenous IL-33 increased tumour size and correlated with ST2^+^ Treg tumour infiltration (95). Overexpression of IL-33 in *Apc* ^min/+^ mice increased tumour burden and correlated with an increase in colonic ST2^+^ Tregs (91), while our group showed that IL-33-deficiency reduced tumour Tregs in *Apc* ^1322T/+^ mice (81), although these studies did not directly assess the functional importance of Tregs. On the other hand, genetic deficiency of IL-25 or rIL-25 treatment did not alter tumour Treg frequency in *Apc* ^1322T/+^ mice (81), suggesting that tumour Tregs preferentially respond to IL-33 and not IL-25 in CRC.

However, whilst Tregs have been implicated in IL-33-mediated CRC tumorigenesis, studies are largely associative and functional depletion studies in the setting of IL-33 perturbation are required. Furthermore, whilst Tregs are often assumed to be pro-tumoral due to their immunosuppressive nature across many cancers (137), the role of Tregs in CRC is more opaque. Certain microbiota are associated with promoting CRC (138, 139), and Tregs may prevent CRC through repressing microbe-induced inflammation (140). In human prognostic studies, a recent systematic review and meta-analysis compiling eight studies found Tregs to be associated with improved survival in human CRC (Odds ratio 0.71, 95% CI 0.62 to 0.82) while correlating with poor survival in most other cancer type assessed (137). In animal models, whilst Treg ablation (DEREG mice) in the AOM/DSS model of CAC reduced tumorigenesis (141), Treg depletion in *Apc* ^min/+^DEREG mice instead increased tumorigenesis (142), supporting the proposal that Tregs may have a protective role in sporadic APC-mutation-mediated CRC. In CAC, the pro-tumoral role of Tregs has been attributed to thymic stromal lymphopoietin (TSLP) rather than IL-33 in one study, as Treg-specific deletion of the TSLP receptor (TSLPR) (*Foxp3* ^IRES-cre^ *Tslpr* ^f/f^) but not ST2 (*Foxp3* ^IRES-cre^ *Il1rl1* ^f/f^) reduced AOM/DSS-mediated CAC (143). While others have found through bone marrow chimera experiments that Treg-specific deletion of ST2 delayed AOM/DSS-mediated CAC disease progression suggesting that Tregs contribute to the pro-tumoral role of IL-33 in CAC (96). A recent immunoprofiling study in human CRC found relative enrichment of Tregs in sporadic CRC compared to CAC (75), consistent with the distinct disease phenotype and immune etiology which may explain the opposite function of Tregs observed. However, as Tregs are heterogeneous with many functionally distinct subsets characterized (144), IL-33-responsive ST2^+^ Treg subsets may potentially promote APC-mutation-mediated CRC and these should be specifically assessed in future studies (87). For now, care must be taken before designating Tregs to be responsible for IL-33-mediated CRC (in particular APC-mutation-mediated CRC) in settings where there is a concomitant rise in tumour Treg infiltration and increased cancer burden, in the absence of functional assessments.

### IL-33 and B cells

Studies have found B cells to inhibit intestinal tumorigenesis in CAC downstream of IL-33. Recently, mice with defects in circadian rhythm (*Bmal1* ^-/-^) were reported to show increased susceptibility to DSS-induced colitis and heightened tumour burden in AOM/DSS-mediated CAC (145). Mechanistically, Bmal1 induces IL-33 expression and IL-33 was found to be necessary to sustain protective intraepithelial PD-L1^+^ regulatory B cells (Bregs). Bregs inhibited pathogenic Th17 cells in an IL-33 and PD-L1-dependent manner thereby suppressing colitis and subsequent CAC. Others have similarly found that genetic IL-33-deficiency results in increased susceptibility to AOM/DSS-mediated CAC (92). In that study, IL-33 promoted B cell production of IgA, which was necessary for microbial homeostasis in the intestines, and defects in the IL-33-B cell-IgA pathway led to dysbiosis characterised by increased mucolytic and colitogenic bacteria, increased IL-1α response, and increased CAC. Cohousing IL-33-deficient and IL-33-replete mice before DSS treatment allowed equilibration of the gut microbiota and abrogated the increased colitis in IL-33-deficient mice seen upon DSS treatment. B cells have also been shown to suppress tumorigenesis in *Apc* ^min/+^ mice similar to in CAC (146). However, currently there are no studies that assessed the role of B cells downstream IL-25 or IL-33 outside CAC.

### IL-25, IL-33 and eosinophils

In the lungs, both IL-25 and IL-33 have been shown to contribute to eosinophil-mediated airway inflammation (147). Currently, studies on eosinophils downstream of IL-25 or IL-33 in CRC mainly focussed on animal models of CAC. Exogenous IL-33 treatment reduced tumour burden in the AOM/DSS model of CAC and correlated with increased eosinophil infiltration (97). Mechanistically, IL-33 treatment enhanced IL-5 and eotaxin-2 expression contributing to eosinophil recruitment. The same study also assessed the role of IL-33 and eosinophils in a heterotypic subcutaneous model of CRC, and found that the anti-tumoral effect of IL-33 was abrogated in mice genetically deficient in eosinophils (ΔdblGATA-1), and adoptive transfer of eosinophils restored the anti-tumour efficacy of IL-33 treatment. Others have found IL-25 to similarly suppress CAC through eosinophils. Antibody-mediated neutralisation of IL-25 increased tumour burden in the AOM/DSS model of CAC and correlated with reduced eosinophils (82), although functional studies were not performed. Therefore, eosinophils may act as a converging node that integrate signals from both IL-25 and IL-33 during intestinal tumorigenesis, particularly CAC. Whether IL-25 and IL-33-mediated eosinophilia occurs in other subtypes of CRC inhibiting tumorigenesis remains to be established, however unlike CAC, most studies are concordant with IL-25 and IL-33 promoting tumorigenesis in APC-mutation-mediated CRC (81, 84, 91). Given that eosinophils are predominantly anti-tumoral in CRC including in *Apc* ^min/+^ mice (148), this could suggest that the IL-25 and IL-33-eosinophil axis may be less prominent in APC-mutation-mediated CRC relative to other pro-tumorigenic pathways compared to in CAC.

## Other pathways modulated by IL-25 and IL-33 in CRC

Due to their broad range of functions IL-25 and IL-33 and can also modulate CRC through additional mechanisms.

### IL-25, IL-33 and cancer stem cells (CSC)

CSCs play important roles in conferring cancer resistance against treatment due to their quiescent nature, and yet are able to regenerate tumours upon insult, and have therefore become attractive therapeutic targets in recent years (149). Both IL-25 and IL-33 have been shown to promote CRC tumour cell stemness. *In vitro* treatment with IL-33 enhanced sphere formation by primary human CRC cells and HT29 cell line, with a concomitant increase in expression of stem cell markers *NANOG*, *NOTCH3*, *OCT3*, *OCT4* and *LGR5* resulting in treatment resistance against 5-fluorouracil in a ST2-dependent manner (94). Others have identified that a small proportion of DCLK1^+^ tuft cells are Lgr5^+^ (stem cell marker) and may act as tumour stem cells during intestinal tumorigenesis, and that DCLK1 expression readily differentiates intestinal tumour stem cells from normal stem cells (80, 150). DCLK1^+^Lgr5^+^ cells can be identified in intestinal tumours that arose following activation of Wnt signaling in Lgr5^+^ stem cells (150), and a recent study confirmed DCLK1 as a downstream target of Wnt signaling (151). DCLK1^+^Lgr5^+^ tumour stem cells resemble tuft cells in their gene expression profile and upregulate other tuft cell markers such as COX-1 (150). IL-25 expression in CRC was reported to be predominantly by DCLK1^+^ tuft-like cells in *Apc* ^1322T/+^ mice (81). Whether it is the acquisition of DCLK1^+^ expression by tumour stem cells due to aberrant Wnt signaling (for example due to loss of APC) which leads to their ability to produce IL-25 remains to be explored. Furthermore, CRC CSCs also express the IL-17BR suggesting potential autocrine signaling (152). Indeed IL-25 may reciprocally promote tumour stemness, as *in vitro* treatment of HT-29 and SW620 human CRC cell lines with IL-25 induced expression of stem markers CD133, Lgr5 and OCT4 through hedgehog signaling, and similarly in the AOM/DSS model of CAC genetic IL-25-deficiency reduced tumour burden and expression of Lgr5, CD133 and DCLK1 (80). Importantly, depletion of DCLK1^+^ tuft cells reduced tumour burden in models of CRC both *in vitro* and *in vivo* (152, 153), indicating that these IL-25-expressing tuft cells can support intestinal tumorigenesis. IL-25 and IL-33 may synergistically promote CRC stemness, indicating that simultaneous blockade of both molecules may be required for maximal therapeutic benefit in attempts to target CSCs.

### IL-25, IL-33 and angiogenesis

Both IL-25 and IL-33 have been implicated in promoting angiogenesis, and angiogenesis mediated by the VEGF family of proteins plays an important and non-redundant role in CRC development and progression, in both patients and mouse models of CRC (154–157). Currently, several VEGF or VEGFR inhibitors (e.g. bevacizumab) are approved by the FDA for treatment of CRC patients (158). In a mouse CRC cell line implant model, transgenic overexpression of IL-33 in MC38 cells (MC38-IL-33) resulted in 3-fold higher microvessel density and increased cancer metastasis to the liver compared to vector control (124). Interestingly, IL-33-mediated angiogenesis in this setting was thought to be VEGF independent, as tumour VEGF expression was lower in MC38-IL-33 tumours compared to control. This suggests that combined blockade of IL-33 and VEGF may potentially further reduce angiogenesis in CRC. Evidence suggests that IL-25 may also promote angiogenesis in CRC. Colonic *Vegfa* expression was found to be reduced upon neutralisation of IL-25, or genetic IL-25-deficiency, in AOM/DSS-treated mice with CAC (82), suggesting that IL-25 may promote tumour VEGF-A expression. Indeed, IL-25 has been reported to induce VEGF expression and promote angiogenesis by human vascular endothelial cells *in vitro* (159), and *in vivo* in a mouse model of asthma where intranasal administration of IL-25 increased VEGF expression and airway vascularity (160). Angiogenesis blockers have proved beneficial clinically against CRC, and combined treatment through the inhibition of IL-25 or IL-33 may provide additional benefits to further reduce tumour angiogenesis.

### IL-25, IL-33 and cancer invasion

IL-25 and IL-33 have been shown to modulate CRC invasion. MMPs are zinc-dependent proteolytic metalloenzymes with roles in degrading extracellular matrix (ECM) proteins. MMP9 is part of the gelatinase family that breakdowns the ECM proteins gelatin and type IV collagen, thereby promoting tumour invasion and metastasis (161, 162). In CRC, increased tumour MMP-9 expression is associated with advanced cancer stages, lymph node metastasis and reduced patient survival (163, 164). However, a study found that genetic deficiency of MMP9 enhanced CAC development and reduced survival in AOM/DSS-treated mice (165) suggesting an anti-tumoral role in CAC in contrast to APC-mutation-driven CRC, where genetic deficiency of MMP9 decreased tumour number by 40% in *Apc* ^min/+^ mice (166). Genetic IL-25 deficiency led to a reduction in tumour *Mmp9* expression in AOM/DSS mice (82), indicating that IL-25 may induce MMP9 expression in CRC. The differential effect of MMP9 in different CRC subtypes may contribute to the opposite effect of IL-25 seen in APC-mutant mice (*Apc* ^1322T/+^) where it promotes tumorigenesis (81), while inhibiting CAC in some studies (82). Interestingly, genetic IL-25-deficiency also led to a reduction in tumour *Mmp2* in AOM/DSS-mediated CAC (82). Similar to MMP9, MMP2 is part of the gelatinase family and is associated with poor overall survival in CRC patients (167). Mechanistically, how IL-25 modulates MMP expression in CRC remains to be established by future studies, but their co-expression may provide additional correlative markers for targeting treatments to specific CRC subtypes.

IL-33 may promote CRC tumour invasion and metastasis through desmoplastic reactions. Desmoplasia during CRC progression is largely mediated by cancer-associated fibroblasts (CAFs) (168), and CRC patients with abundant desmoplasia are more likely to have lymphatic metastases (169). CAFs in human CRC express IL-33, and IL-33 expression levels in CAFs correlated with the degree of human CRC cell line migration when treated with CAF conditioned media (169). In the metastatic lymph node from human CRC patients, IL-33 expression is detected in CAF-like cells at areas of high desmoplasia at the tumour invasive front. In addition, CRC epithelial IL-33 expression was also found to correlate with increased desmoplasia, indicating that both epithelial and CAF-derived IL-33 can promote CRC desmoplasia, invasion and metastasis. Mechanistically, IL-33 promotes epithelial mesenchymal transition (EMT) characterised by decreased E-cadherin and increased vimentin and N-cadherin expression in HT29 human CRC cells, and induced migration in a wound healing assay *in vitro*. In another study, IL-33 stimulation of primary human CRC cells increased expression of MMP2 and MMP9, and shRNA-mediated knockdown of ST2 or inhibition of MMP2 and MMP9 reduced invasion *in vitro* (170). Therefore, both IL-25 and IL-33 may promote CRC invasion through induction of MMPs. Conversely, others have found that IL-33 may instead inhibit metastasis through inducing CD40L expression on tumour infiltrating lymphocytes (171). Here, rIL-33 treatment reduced pulmonary and liver metastases by CT26 cells when injected *via* the tail vein and into the splenic capsule respectively, and concomitant anti-CD40L treatment significantly promoted tumour burden and reduced IFNγ expression in CD4^+^ T cells, NK cells and CD8^+^ T cells in IL-33 treated mice. Altogether, these studies suggest functional divergence of IL-33 when acting on tumour cells and the immune infiltrate, where direct action of IL-33 on tumour cells induces desmoplasia and tumour invasion, while IL-33-mediated stimulation of tumour infiltrating lymphocytes may conversely limit CRC metastasis. Therefore, the overall effect of IL-33 on CRC metastasis may possibly depend on the relative immunogenicity of the different CRC subtypes.

## Future perspectives - IL-25 and IL-33 in cancer immunotherapy

In the past decade checkpoint inhibitors have shown unprecedented efficacy against a broad range of cancer types (172). In the tumour microenvironment where antigen stimulation persists, effector T cells proceed to functional exhaustion (173). These exhausted T cells have significantly impaired effector functions, and are characterised by increased and sustained expression of an array of inhibitory checkpoint receptors, the best studied being cytotoxic T-lymphocyte-associated protein 4 (CTLA-4), programmed cell death protein 1 (PD-1) and lymphocyte-activation gene 3 (LAG-3) (174, 175). Ligation with their cognate ligands, expressed on tumour cells, effectively blunts T cell effector function and promotes tumour survival (174). This can be reversed clinically through the use of checkpoint inhibitors, e.g. monoclonal antibodies that block the inhibitory receptor-ligand interaction between T cells and tumours, thereby revitalizing exhausted T cells to effectively mediate anti-tumour functions (176, 177). However despite their great success, checkpoint inhibitors are currently largely ineffective in human MSS CRC, which show a less robust T cell response compared to MSI-high CRC (4, 63, 64, 178). Importantly, MDSCs have been shown to play critical roles in treatment-resistance across several modalities of cancer treatment including chemotherapy, radiotherapy and immunotherapy (checkpoint inhibitors) in a broad range of cancers including CRC (4, 179–182). Mechanistically, MDSCs suppress anti-tumoral T cells through multiple pathways (**Figure 5**) thereby limiting the efficacy of checkpoint inhibitors. Antibody-mediated blockade of IL-25 signaling effectively reduced immunosuppressive tumour M-MDSCs and enhanced IFNγ expression in tumour infiltrating CD4^+^ and CD8^+^ T cells resulting in reduced tumour burden in *Apc* ^1322T/+^ mice (81). This raises the possibility that concomitant IL-25-signalling blockade may potentiate checkpoint inhibitor treatment through suppressing MDSCs thereby enhancing immunotherapeutic efficacy against CRC. This could be coupled with using IL-25 as a biomarker, in particular in CRC patients with high tumour IL-25 expression, as elevated tumour IL-25 is associated with poor survival in CRC patients (80, 81) and these patients may be more responsive to IL-25 signaling blockade. Similarly, IL-33 may promote intestinal tumorigenesis in the *Apc* ^min/+^ model potentially through immunosuppressive ST2^+^ Tregs and mast cells (84, 91) and IL-33 blockade may similarly liberate anti-tumour immunity and improve checkpoint inhibitor efficacy. In particular, blocking IL-33 may allow preferential depletion of the likely pro-tumoral ST2^+^ Treg subset, which may prove to be more beneficial than other methods that target Tregs nonspecifically, especially as unlike in most cancers Tregs are associated with good prognosis in human CRC patients (137).

Conversely, conflicting reports have shown that both IL-33 and IL-25 may either promote or inhibit CAC (**Table 1**) (80, 82, 90, 92, 96, 97), and therefore caution should be taken when considering IL-25 or IL-33-based therapy in this cohort. Another strategy would be to target IL-25 or IL-33 during early stages of IBD, to delay or prevent progression into intestinal cancer while resolving the colitis. This would be particularly useful in the management of Crohn’s disease (CD) as unlike ulcerative colitis (UC), CD affects the whole alimentary tract and cannot be cured through pancolectomy. Nevertheless, early treatment in UC may improve patient quality of life and reduce morbidity through delaying the need for colectomy and the requirement of a stoma. A recent study found that in UC patients, there is decreased expression of 8 miRNAs of the miR-378 family which targets IL-33 mRNA, and is associated with an almost 4-fold increase in IL-33 mRNA. Of these, miR-378a-3p was further decreased in active UC patients compared those under remission and overexpression of this miRNA reduced IL-33 expression *in vitro* (183). Importantly, both IL-33 and IL-25 have been shown to have dual roles in IBD similar to in CAC (184, 185), and therefore miRNA-based therapy may become an attractive therapeutic option in the future to fine-tune IL-33 or IL-25 expression levels in the management of colitis and CRC in different subsets of patients.

**Table 1:** Summary of animal studies illustrating the pro and anti-tumorigenic roles of IL-25 and IL-33 in different models of CRC.

Study	CRC subtype/model	IL-25 role	IL-33 role	Proposed mechanism
Jou et al. (81)	*Apc* ^1322T/+^ model of APC-mutation-mediated CRC	Promotes tumorigenesis; genetic deficiency of IL-25 or antibody-mediated blockade of IL-17BR reduced tumour burden	Promotes tumorigenesis; genetic deficiency of IL-33 reduced tumour burden	IL-25 promotes tumorigenesis through activating ILC2s which sustain tumour M-MDSCs to suppress T cell and IFNγ-mediated anti-tumour immunity. IL-33 deficiency is associated with a reduction in tumour Tregs and mast cells.
Thelen et al. (82)	AOM/DSS model of CAC	Inhibits tumorigenesis; antibody-mediated blockade of IL-25 increased tumour burden	Not assessed	IL-25 blockade led to increased colitis and correlated with reduced colonic eosinophils.
Liu et al. (80)	AOM/DSS model of CAC	Promotes tumorigenesis; genetic deficiency of IL-25 reduced tumour burden	Not assessed	IL-25 promotes tumorigenesis through maintaining tumour stemness, and genetic IL-25-deficiency led to reduced expression of stem cell markers Lgr5, CD133 and DCLK1.
Benatar et al. (83)	Subcutaneous heterotypic HT29 implant in CD1 athymic nude mice	Inhibits tumorigenesis; exogenous IL-25 treatment reduced tumour burden	Not assessed	Anti-tumour effect of IL-25 is B cell dependent as only observed in CD1 athymic nude mice but not in SCID mice.
Maywald et al. (84)	*Apc* ^min/+^ model of APC-mutation-mediated CRC	Not assessed	Promotes tumorigenesis; genetic deficiency of IL-33 or antibody-mediated blockade of ST2 reduced tumour burden	IL-33 deficiency or blockade led to reduced tumour *Il4*, *Il6* and *Il13* expression, and correlated with reduced tumour mast cells.
He et al. (91)	*Apc* ^min/+^ model of APC-mutation-mediated CRC	Not assessed	Promotes tumorigenesis; genetic overexpression of IL-33 in epithelial tumour cells increased tumour burden	Genetic overexpression of IL-33 in tumour epithelial cells is associated with an increase in colonic alternative activated macrophages and ST2^+^ Tregs.
Mertz et al. (90)	AOM/DSS model of CAC	Not assessed	Promotes tumorigenesis; genetic deficiency of ST2 reduces colitis and tumour burden	ST2-dependent IL-33 signalling disrupts intestinal barrier integrity resulting in increased serum LPS and IL-6 induction consistent with enhanced colitis.
Pastille et al. (96)	AOM/DSS model of CAC	Not assessed	Promotes tumorigenesis; ST2^+^ Tregs in colon positively correlated with tumour burden	IL-33 suppresses IL-17 production by ST2^+^ Tregs, prevents Treg polarization towards a Th17 phenotype, and promotes Treg-mediated suppression of CD8^+^ T cells.
Malik et al. (92)	AOM/DSS model of CAC	Not assessed	Inhibits tumorigenesis; genetic IL-33 deficiency increased colitis and tumorigenesis	IL-33 promotes intestinal IgA production by B cells which is required for intestinal homeostasis and prevention of colitis.
Liu et al. (145)	AOM/DSS model of CAC	Not assessed	Inhibits tumorigenesis; genetic deficiency of the Bmal1-IL-33 pathway increased tumour burden	IL-33 promotes intestinal intraepithelial PD-L1^+^ Breg cells which sustains intestinal homeostasis and prevents colitis.
Kienzl et al. (97)	AOM/DSS model of CAC	Not assessed	Inhibits tumorigenesis; IL-33 treatment reduced tumour burden	IL-33 treatment reduced tumour burden and is associated with an increase in eosinophil gene expression in tumours.
	Subcutaneous heterotypic CT26 implant model			IL-33-mediated suppression of tumorigenesis was eosinophil dependent, as genetic deficiency of eosinophils abrogated the anti-tumoral effects of IL-33 which is restored upon adoptive transfer of eosinophils.
Li et al. (93)	Subcutaneous heterotypic MC38 implant model	Not assessed	Promotes tumorigenesis; MC38 implant in IL-33 transgenic mice increased tumour burden compared to wild-type control	IL-33 promotes tumour growth and proliferation in a COX-2 dependent manner, which is reversed upon ST2 blockade or COX-2 inhibition.
Zhang et al. (124)	Orthotopic caecal MC38 implant model	Not assessed	Promotes tumorigenesis; overexpression of IL-33 in MC38 tumour cells increased metastasis to the liver	Overexpression of IL-33 in tumour cells is associated with increased recruitment of tumour MDSCs and enhanced angiogenesis.
Zhou et al. (95)	Subcutaneous heterotypic CT26 implant model	Not assessed	Promotes tumorigenesis; exogenous IL-33 treatment increased tumour burden while antibody-mediated blockade of IL-33 signalling reduced tumour burden	IL-33 treatment enhanced tumour burden and correlated with an increase in tumour ST2^+^ Tregs.
Fang et al. (94)	Subcutaneous heterotypic MC38 implant model	Not assessed	Promotes tumorigenesis; MC38 implant in IL-33 transgenic mice showed increased tumour growth compared to control	IL-33 promotes colon cancer stemness. IL-33 treatment *in vitro* increased HT29 and primary human CRC cell expression of stem markers NANOG, NOTCH3, OCT3, OCT4 and Lgr5, and enhanced sphere formation.
	Subcutaneous heterotypic primary human CRC implant model		Promotes tumorigenesis; human primary CRC implants in nude mice showed increased tumour growth when treated with exogenous IL-33	
Luo et al. (171)	Subcutaneous heterotypic CT26 and MC38 implant model	Not assessed	Inhibits tumorigenesis; exogenous IL-33 treatment reduced CT26 and MC38 tumour growth and metastasis	IL-33 induces CD40L expression by tumour infiltrating lymphocytes which promotes IFNγ^+^ production by NK cells and T cells.

Finally, IL-33 and IL-25 contribute to many processes involved with CRC pathogenesis, for example in shaping the intestinal immune milieu and CRC tumour microenvironment, as well as their involvement in angiogenesis, desmoplasia, tumour invasion, metastasis and in sustaining CSCs. This broad range of functions opens avenues for potential combination therapies with other existing cancer treatments, such as chemotherapy, radiotherapy or targeted therapy. For instance, chemotherapy-induced cancer cell death results in release of tumour antigens (186), and concomitant IL-25 blockade may inhibit MDSCs and liberate anti-tumour T cells to recognize and eliminate tumours. On the other hand, IL-33 promotes angiogenesis in CRC in a VEGF-independent manner (124), and therefore simultaneous blockade of IL-33 together with VEGF inhibitors (currently used clinically) may show potentiation. In the metastatic setting, targeting IL-33 and IL-25 may also prove beneficial given their role in CRC invasion and metastasis, and may also potentially be combined with existing therapies in the future for metastatic disease.

## Conclusions

Due to their prominent role in regulating the intestinal immune response, IL-33 and IL-25 have become attractive targets in CRC. Emerging studies in the past decade have found IL-33 and IL-25 to either promote or inhibit CRC under different settings (**Figures 3** and **4**), and is likely dependent on the CRC subtype, tumour immunogenicity and the different immune cell populations involved (**Table 1**). Therefore, it is important to understand the context in which immune reactions arise in CRC and how they may differ substantially between the distinct CRC subtypes (75). As evidenced by the varying roles of IL-25 and IL-33 in different CRC subtypes and mouse models, it is critical to understand how such innate signals contribute to disease diversity. Furthermore, the additional broad range of actions of IL-33 and IL-25 affecting multiple pathways associated with CRC pathogenesis such as angiogenesis and metastasis would mean that the overall effect of these cytokines would depend on many factors including the relative contribution of each pathway in different CRC subtypes. Future advances in novel CRC models that more closely reflect the human CRC immune environment for different CRC subtypes and microbiome constitution, will further refine our understandings of IL-25 and IL-33 in CRC, and facilitate therapeutic development. Although current immunotherapeutic strategies largely rely on stimulating or engineering adaptive T cells and generally lack efficacy in CRC, it may be possible to target such upstream innate immune signals to improve therapeutic efficacy in defined subtypes. Identifying the relevant factors that govern anti-tumour immunity in specific cancer subtypes may therefore open-up novel more refined opportunities for future immunotherapy. Finally, uncovering novel biomarkers in CRC patients that predict therapeutic response to targeted therapy against IL-25 or IL-33 will be crucial in facilitating their introduction into the clinic for treating CRC in the future.

## Author contributions

All authors listed have made a substantial, direct, and intellectual contribution to the work, and approved it for publication.

## Funding

This work was supported by the Medical Research Council, as part of United Kingdom Research and Innovation (also known as UK Research and Innovation) U105178805.

## Acknowledgments

We thank the McKenzie lab for feedback on this review article. Parts of **Figure 1**, **2**, **4** and **5** were created using cartoon templates by Servier Medical Art, licensed under a Creative Commons Attribution 3.0 Unported License (https://smart.servier.com/).

## Conflict of interest

ANJM is on the scientific advisory board of SinoMab. ANJM developed antibodies against IL-25R which MRC has licenced to SinoMab.

The remaining authors declare that the research was conducted in the absence of any commercial or financial relationships that could be construed as a potential conflict of interest.

## Publisher’s note

All claims expressed in this article are solely those of the authors and do not necessarily represent those of their affiliated organizations, or those of the publisher, the editors and the reviewers. Any product that may be evaluated in this article, or claim that may be made by its manufacturer, is not guaranteed or endorsed by the publisher.
